# Comparison of pancreaticoduodenectomy and bile duct resection for middle bile duct cancer: A multi‐center collaborating study of Japan and Korea

**DOI:** 10.1002/jhbp.724

**Published:** 2020-03-11

**Authors:** Hiroki Hayashi, Jin‐Young Jang, Kyung Sik Kim, Jin Sub Choi, Takeshi Takahara, Sung Hoon Choi, Satoshi Hirano, Hee Chul Yu, Syuichiro Uemura, Michiaki Unno

**Affiliations:** ^1^ Department of surgery Tohoku University Graduate School of Medicine Sendai Japan; ^2^ Department of Surgery Seoul National University College of Medicine Seoul Korea; ^3^ Department of Surgery Yonsei University College of Medicine Seoul Korea; ^4^ Department of Surgery Iwate Medical University Morioka Japan; ^5^ Department of Surgery CHA University Pocheon Korea; ^6^ Department of Gastroenterological Surgery II Hokkaido University Faculty of Medicine Sapporo Japan; ^7^ Department of Surgery Chonbuk National University Medical School Jeonju Korea; ^8^ Institute of Gastroenterology Tokyo Women's Medical University Tokyo Japan

**Keywords:** middle bile duct cancer, pancreaticoduodenectomy, bile duct segmental resection, extrahepatic cholangiocarcinoma

## Abstract

**Background:**

It is currently unknown whether bile duct segmental resection (BDSR) is an acceptable method for localized middle bile duct cancer (mid‐BDC) when R0 resection can be achieved. This study aimed to investigate the short‐ and long‐term outcomes of mid‐BDC patients treated with pancreaticoduodenectomy (PD) compared to those for BDSR.

**Methods:**

This was a retrospective, Japanese and Korean multi‐center collaboration study based on patients' medical records.

**Results:**

A total of 663 patients, including 245 BDSR and 418 PD cases, were enrolled. The incidence of postoperative pancreatic fistula (3.3% vs 44.1%, *P* < .0001), surgical site infection in the organ space (6.1% vs 17.7%, *P* < .0001) and clinically problematic morbidities (15.9% vs 32.8%, *P* < .0001) was significantly higher in the PD group. There was no difference in the mortality rate (0.8% vs 1.7%, *P* = .3566). Local (33.9% vs 14.4%, *P* < .0001) and lymph node (22.4% vs 11.0%, *P* < .0001) recurrence rates were significantly higher in the BDSR group. Relapse‐free survival (25.0 vs 34.0 months, *P* = .0184) and overall survival (41.2 vs 60.1 months, *P* = .0019) were significantly longer in the PD group. The PD group had significantly better prognosis in stage IA/IB cases (58.3 vs 111.5 months, *P* = .0067), which were the best indicators for BDSR, even when R0 resection was achieved. In multivariate analysis, BDSR was an independent poor prognostic factor.

**Conclusion:**

Despite the inferior perioperative short‐term outcomes, our data advocate that PD should be the standard procedure for mid‐BDCs and that BDSR should be avoided even if R0 resection can be achieved. (UMIN000017914).

## Graphical abstract

***Highlight***

This retrospective Japanese and Korean multicenter collaborative study investigated the short‐ and long‐term outcomes of patients with localized middle bile duct cancer, comparing those treated with pancreaticoduodenectomy with those treated with bile duct segmental resection. Hayashi and colleagues concluded that pancreaticoduodenectomy should be the standard procedure for middle bile duct cancer.

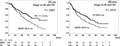

## 1 INTRODUCTION

Curative resection without any cancer remnants (R0 resection) is considered to be essential for the cure of distal bile duct cancer (BDC). Moreover, the presence of regional lymph node metastasis and pancreatic invasion are reported to be poor prognostic factors for distal BDC.^1^, ^2^, ^3^, ^4^ Pancreaticoduodenectomy (PD), also known as the Whipple procedure, is necessary to achieve R0 resection in distal BDC patients with pancreatic invasion or with cancer metastases to the lower bile duct. PD is considered to be the standard procedure for middle BDC (mid‐BDC), defined as Bm in the General Rules for Surgical and Pathological Studies on Cancer of the Biliary Tract (Japanese Society of Biliary Surgery),^5^, ^6^ as well as distal BDC. However, in some cases of localized mid‐BDC without pancreatic invasion and extension to the lower and upper bile duct, R0 resection can be achieved with bile duct segmental resection (BDSR) alone without pancreatectomy and hepatectomy.

A Korean group reported the short‐term and long‐term outcomes for patients who underwent BDSR or PD for localized mid‐BDC.^7^ In their report, the survival curve of patients in the BDSR group was equivalent to that of the PD group, and a lower incidence of postoperative morbidity along with shorter postoperative hospital stay were observed in the BDSR group than in the PD group. The authors of this study concluded that BDSR could be justified as an alternative radical operation for mid‐BDC in a select group of patients with no adjacent organ invasion and negative resection margin. Another Korean group reported that, though the R0 resection rate was higher in the PD group than in the BDSR group, there was no significant difference in the prognosis of mid‐BDC patients between the BDSR and PD groups when R0 resection was achieved. They also concluded that BDSR could be applied only when R0 resection was possible or if the patients had comorbidities that precluded PD.^8^

Based on these previous reports, if BDSR is equivalent to PD in terms of long‐term survival and if BDSR has a lower rate of morbidity than PD, BDSR should be performed first for mid‐BDC cases, and if the margins of the proximal and distal bile duct cut end are negative, BDSR alone is necessary and sufficient as a curative procedure. If the distal margin is positive, then PD should be additionally performed for R0 resection. In order to establish this treatment strategy, it is necessary to show that the prognosis of BDSR is equal to or better than that of PD, and it is also necessary to show that the prognosis of additionally performed PD (AdPD) is equal to or better than that of PD alone when the distal bile duct cut margin is positive.

In these previous reports, BDSR was performed in as few as 45 cases and 43 cases, respectively, and the equivalence of BDSR to PD could not be concluded because of the small number of cases. Hence, this study was designed to accumulate further cases from Japan and Korea and to verify whether BDSR for mid‐BDC would be acceptable as the standard procedure. In this study, AdPD cases were excluded because of their small number, which was insufficient for statistical consideration; thus, BDSR and PD were compared.

This study was conducted as the biliary part of a collaboration study between Japan and Korea based on the proposal by the Japanese Society of Hepato‐Biliary‐Pancreatic Surgery.

## 2 METHODS

### 2.1 Participating facilities

Under the approval of the research ethics committee or the institutional review board at each facility, 40 facilities from Japan and nine facilities from Korea participated in this study. This retrospective study was approved by the institutional review board of the Tohoku University School of Medicine (approval number: 2014‐1‐779) and at the ethics committee of each institution.

### 2.2 Patients

Among the patients with mid‐BDC, with ECOG performance status was 0 or 1, and age between 20 and 80 years, who underwent BDSR or PD in the participating institutions between 2001 and 2010, preoperatively diagnosed with clinically T4; T3 with hepatic invasion; and M1 cases according to the UICC 7th edition, were excluded. Further, 48 cases of pathologically T4 (pT4) and 96 cases of pM1 were also excluded. In addition, patients requiring hepatectomy, patients with preoperative chemo (radio)‐therapy, and patients who had <5 years since the diagnosis and treatment of other malignant diseases were excluded.

Based on the above criteria, a total of 663 cases, 364 from Japan and 299 from Korea, were enrolled in this study. All facilities and the number of cases are listed in the acknowledgments.

### 2.3 Survey method

A questionnaire analysis based on medical records was conducted and anonymized at each participating facility. These data were compiled and analyzed at the Department of Surgery, Tohoku University school of Medicine.

### 2.4 Outcome measures

Sex, TNM stage (the 7th edition UICC TNM classification^9^), and differentiation of each operative procedure (BDSR or PD) were analyzed. The presence of lymphatic invasion (ly), venous invasion (v), (peri‐) neural invasion (ne), portal system invasion (PV), residual tumor (R) of the proximal bile duct margin (HM), distal bile duct margin (DM) and dissected margin (EM) by the classification of biliary tract cancers established by the Japanese Society of Hepato‐Biliary‐Pancreatic Surgery: 3rd English edition^10^ were also evaluated.

The duration of postoperative hospital stays, incidence of mortality and postoperative morbidities such as the grade of postoperative pancreatic fistula (POPF) defined by ISGPF,^11^ anastomotic leakage, postoperative hemorrhage, and surgical site infection (SSI) were evaluated as short‐term outcomes. Overall survival time (OS), relapse‐free survival time (RFS) and incidence of recurrent at each site were evaluated as long‐term outcomes.

### 2.5 Statistical analysis

Statistical analyses were performed using the JMP Pro 13 software (SAS Institute Inc.). The data distribution was tested for normality by examining the mean and standard error. Continuous variables are reported as median values and 25–75th percentile interquartile ranges (IQR). Comparisons were performed using the Wilcoxon rank‐sum test for non‐normally distributed continuous variables. Chi‐squared and Fisher's exact tests (in cases of low frequencies) were used for comparisons of categorical variables. The relative risk and 95% confidence interval (CI) are also reported. The Kaplan–Meier method was used for comparison of OS and RFS and the log‐rank test was used to compare the statistical survival distributions between the two groups.

## 3 RESULTS

The patients' backgrounds and the histopathological results of patients with BDSR and PD are shown in Table 1. The proportion of BDSR cases was high in Korea, while that of PD was high in Japan. There were no differences in age distribution or gender ratios between the BDSR and PD groups. The proportion of patients with pathological T2 (pT2) cancer and below was higher in the BDSR group, and the proportion of the patients with pT2 and above was higher in the PD group; the rate of pT2 cases was highest in both groups (BDSR 60.4%, PD 44.7%). Although there was no statistical difference, the ratio of cases with lymph node metastasis (pN1) was higher in the PD group. Accordingly, PD tended to be performed for more advanced cases. This tendency was similar in both Japan and Korea. There was no difference in the degree of histological differentiation in each group, but the ratio of cases with positive ly, v, and ne was significantly higher in the PD group (Table 1).

**Table 1:** The differences in patients' background and clinicopathological features in the BDSR and PD groups

	BDSR^d^ n = 245	PD n = 418	*P*
Age			
Median	69	68	.2371
25%‐75% IQR	62‐74	62‐73	
Sex			
Female	80 (32.7%)	121 (28.9%)	.3163
Male	165 (67.3%)	297 (71.1%)	
Country			
Japan	94 (38.4%)	270 (64.6%)	<.0001
Korea	151 (61.6%)	148 (35.4%)	
pT			
1	63 (25.7%)	59 (14.1%)	<.0001
2	148 (60.4%)	187 (44.7%)	
3	34 (13.9%)	172 (41.1%)	
pN			
0	175 (71.4%)	270 (64.6%)	.0706
1	70 (28.6%)	148 (35.4%)	
pStage			
1A	59 (24.1%)	53 (12.7%)	<.0001
IB	103 (42.0%)	124 (29.7%)	
2A	13 (5.3%)	93 (22.2%)	
2B	70 (28.6%)	148 (35.4%)	
Differentiation			
Well	72 (30.4%)	110 (27.9%)	.3617
Mod.	119 (50.2%)	214 (54.3%)	
Poor	45 (19.0%)	70 (17.8%)	
Undiff.	1 (0.4%)	0 (0.0%)	
v^a^			
−	156 (69.3%)	216 (55.4%)	.0007
+	69 (30.7%)	174 (44.6%)	
ly^b^			
−	124 (55.1%)	161 (41.3%)	.0009
+	101 (44.9%)	229 (58.7%)	
ne^c^			
−	73 (30.7%)	96 (23.6%)	.0483
+	165 (62.0%)	311 (57.6%)	

^a^ Venous invasion.

^b^ Lymphatic invasion.

^c^ (Peri‐) neural invasion.

^d^ Bile duct segmental resection.

A comparison of the degree of surgical invasion, curability, and the incidence of postoperative complications in each procedure is shown in Table 2. Compared to BDSR, the operation time was significantly longer in the PD group (median: BDSR 423 minutes, PD 517 minutes, *P* < .0001) and the intraoperative bleeding volume was also significantly larger in the PD group (median: BDSR 607 mL, PD 1020 mL, *P* < .0001). However, the rate of R0 resection was significantly higher in the PD group (BDSR 55.5%, PD 68.4%, *P* = .0022). It is not surprising that the rate of negative distal bile duct margin (DM) was higher in the PD group (BDSR 73.5%, PD 100%, *P* < .0001), but the rate of negative proximal bile duct margin (HM) was also significantly higher (BDSR 74.3%, PD 81.3%, *P* = .0234). In addition, there was a strong tendency for a higher negative ratio of the dissected margin (EM) (BDSR 76.3%, PD 82.8%, *P* = .0754), as well as higher curability in the PD group. As the number of T factors progressed, the R0 ratio decreased particularly in the BDSR group, and the R0 ratio of the BDSR group was significantly lower than that of the PD group in T2 (BDSR 56.1%, PD 67.4%, *P* = .0340) and T3 (BDSR 35.3%, PD 66.3%, *P* = .0072) cases. The total number of retrieved lymph nodes was significantly smaller in the BDSR group than in the PD group (median: 8 vs 17, *P* < .0001).

**Table 2:** Comparison of surgical invasion, curability, and postoperative morbidity between the BDSR and PD groups

	BDSR	PD	*P*
	n = 245	n = 418	
Operation duration (min)			
Median	423	517	<.0001
25%‐75% IQR	350‐506	436‐615	
Blood loss (mL)			
Median	607	1020	<.0001
25%‐75% IQR	395‐1028	603‐1440	
Hospital stay after operation (d)			
Median	18	33	<.0001
25%‐75% IQR	13‐26	23‐47	
R^a^			
0	136 (55.5%)	286 (68.4%)	.0022
1	78 (31.8%)	102 (24.4%)	
2	31 (12.7%)	30 (7.2%)	
R0 ratio in each T			
T1	65.1%	78.0%	.1158
T2	56.1%	67.4%	.0340
T3	35.3%	66.3%	.0007
R0 ratio in N0/1 cases			
N0	61.7%	73.3%	.0098
N1	40.0%	59.5%	.0072
Number of retrieved LNs			
Median	8	17	<.0001
25%‐75% IQR	4‐14	10‐26	
HM^b^			
0	182 (74.3%)	340 (81.3%)	.0234
1	52 (21.2%)	55 (13.2%)	
2	11 (4.5%)	23 (5.5%)	
DM^c^			
0	180 (73.5%)	418 (100.0%)	<.0001
1	51 (20.8%)	0 (0.0%)	
2	14 (5.7%)	0 (0.0%)	
EM^d^			
0	187 (76.3%)	346 (82.8%)	.0754
1	47 (19.2%)	63 (15.1%)	
2	11 (4.5%)	9 (2.2%)	
POPF^e^			
‐	237 (96.7%)	233 (55.9%)	<.0001
+	8 (3.3%)	184 (44.1%)	
POPF^e^ grade			
None	237 (96.7%)	233 (57.1%)	<.0001
A	1 (0.4%)	46 (11.3%)	
B	5 (2.0%)	111 (27.2%)	
C	2 (0.8%)	18 (4.4%)	
Biliary fistula			
−	233 (95.1%)	407 (97.4%)	.1237
+	12 (4.9%)	11 (2.6%)	
GI tract leakage			
−	242 (98.8%)	406 (97.1%)	.1688
+	3 (1.2%)	12 (2.9%)	
Hemorrhage			
−	238 (97.1%)	394 (95.2%)	.4367
+	7 (2.9%)	20 (4.8%)	
SSI^f^ (organ space)			
−	230 (93.9%)	344 (82.3%)	<.0001
+	15 (6.1%)	74 (17.7%)	
Clavien‐Dindo grade^g^			
0, 1, 2	206 (84.1%)	281 (67.2%)	<.0001
3a	32 (13.1%)	111 (26.6%)	
3b, 4a, 4b, 5	7 (2.9%)	26 (6.2%)	
Mortality			
−	243 (99.2%)	411 (98.3%)	.3566
+	2 (0.8%)	7 (1.7%)	

^a^ The extent of residual cancer after surgery.

^b^ Proximal margin of bile duct (hepatic side).

^c^ Distal bile duct margin.

^d^ Dissected margin.

^e^ Post‐operative pancreatic fistula: an international study group of pancreatic fistula.

^f^ Surgical site infection.

^g^ Clavien‐Dindo Classification of Surgical Complications.

Although there were no statistically significant differences in the incidence of biliary fistula, gastrointestinal anastomosis leakage, and postoperative hemorrhage between the two procedures, the incidence of POPF (BDSR 3.3%, PD 44.1%, *P* < .0001) and the incidence of SSI in the organ space (BDSR 6.1%, PD 17.7%, *P* < .0001) were significantly higher in the PD group. As a result, postoperative hospital stay was also significantly longer in the PD group (BDSR 18 days, PD 33 days, *P* < .0001) (Table 2). This trend did not differ between Japan (BDSR 22 days, PD 40 days, *P* < .0001) and Korea (BDSR 16 days, PD 23.5 days, *P* < .0001) despite the different healthcare systems.

**Figure 1:**
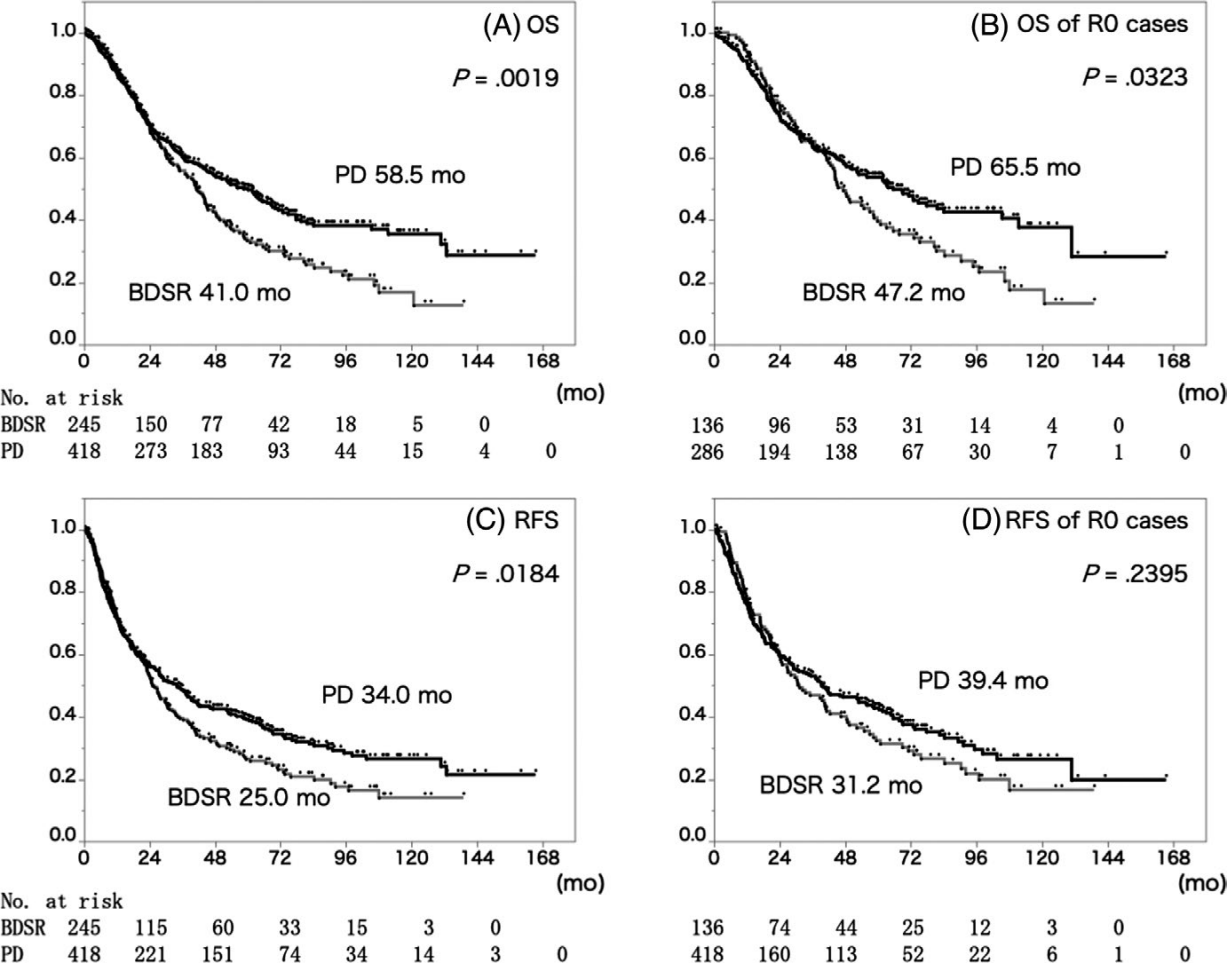
Overall survival of all the patients (A) and the patients with R0 resection (B), and relapse‐free survival of all the patients (C) and the patients with R0 resection (D). Gray line and black line show the survival curve of the patients who received BDSR and PD, respectively. Regardless of whether R0 resection was obtained or not, OS was significantly longer in the PD group. There were no significant differences between the PD group and the BDSR group when R0 resection was achieved

Though PD tended to be performed in more advanced and aggressive mid‐BDC patients, the MST and RFS of the PD group were significantly longer than those of the BDSR group (MST: BDSR 41.0 months, PD 58.5 months, *P* = .0019) (RFS: BDSR 25.0 months, PD 34.0 months, *P* = .0184). Even within the R0 resected cases (BDSR n = 136; PD n = 286), the MST was significantly longer in the PD group (BDSR 47.2 months, PD 65.5 months, *P* = .0323), but there was no statistical difference in the RFS (BDSR 31.2 months, PD 39.4 months, *P* = .2395) (Figure 1).

The MST stratified according to pT stages was also significantly longer in the PD group at any pT stage (pT1: BDSR 58.3 months, PD not achieved, *P* = .0029; pT2: BDSR 37.0 months, PD 56.2 months, *P* = .0029; pT3: BDSR 25.3 months, PD 34.6 months, *P* = .0147). Strikingly, the MST of cases in the PD group was significantly longer than that of the BDSR group regardless of the presence of regional lymph node metastasis (pN0: BDSR 51.2 months, PD 77.7 months, *P* = .0042; pN1: BDSR 25.3 months, PD 29.2 months, *P* = .0091). Moreover, the MST of the PD group was significantly better for Stages IA, IB, and IIB (pStage IA: BDSR 59.7 months, PD not achieved, *P* = .0019; Stage IB: BDSR 45.3, PD 67.1 months, *P* = .0313; pStage IIB: BDSR 27.9 months, PD 30.3 months, *P* = .0091). There was no significant difference in pStage IIA (T3N0M0), perhaps due to a small number of BDSR cases, but a favorable prognostic tendency was observed in the PD group (BDSR 27.8 months, PD 52.9 months, *P* = .1296) (Table 3).

**Table 3:** Univariate and multivariate analysis of prognostic factors of mid‐BDC

	n	MST (mo)	Univariate *P*	Multivariate *P*	HR	95% CI
pT						
3	206	32.6	.0003	.0159	1.36	1.06‐1.74
1, 2	457	56.9				
pN						
1	218	27.9	<.0001	<.0001	1.84	1.44‐2.35
0	445	65.5				
v^a^						
+	243	37.9	.0387	.1541		
−	372	53.4				
ly^b^						
+	330	40.7	.0014	.6164		
−	285	63.0				
ne^c^						
+	476	41.0	<.0001	<.0001	1.86	1.36‐2.53
−	169	96.9				
R^d^						
1, 2	241	34.7	.0002	.0286		
0	422	58.3				
Differentiation						
Non‐well	449	39.3	<.0001	.0002	1.67	1.26‐2.21
Well	182	94.7				
Numbers of retrieved LNs						
<13	312	40.3	.003	.1579		
≥13	336	59.7				
Procedure						
BDSR	245	41.2	.0019	.0010	1.57	1.2‐2.05
PD	418	60.1				

^a^ Venous invasion.

^b^ Lymphatic invasion.

^c^ (Peri‐) neural invasion.

^d^ The extent of residual cancer after surgery.

**Figure 2:**
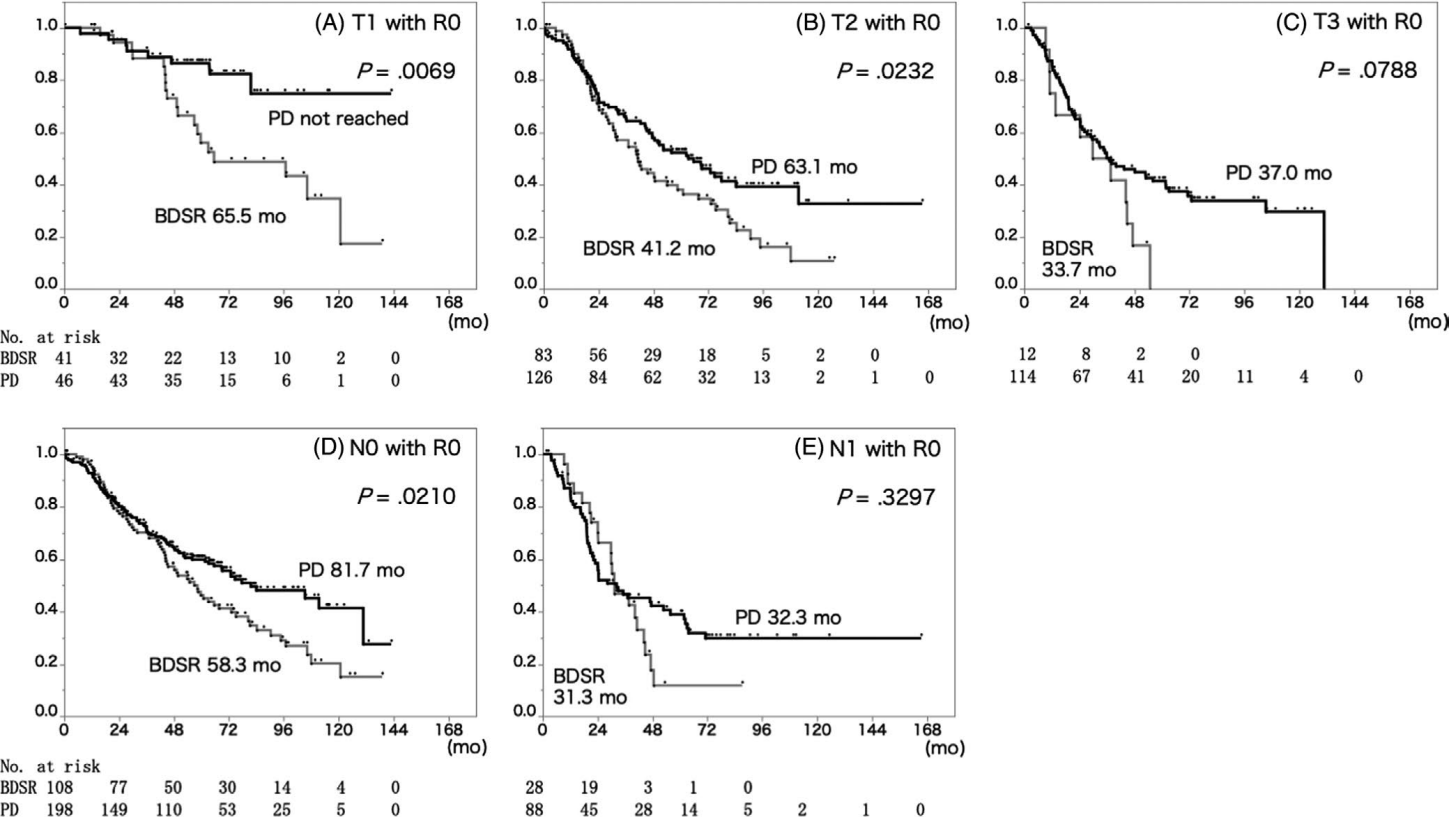
Survival curves of the patients with R0 resection stratified by T factor and N factor. Cox‐regression survival probability of (A) T1, (B) T2, (C) T3, (D) N0 and (E) N1 patients who received BDSR (gray line) or PD (black line). In T1, T2 and N0 cases, the PD group had significantly longer survival compared to the BDSR group

Even with a limited number of cases in which R0 resection was achieved, the PD group had a significantly better prognosis in T1 (MST; BDSR 65.5 months, PD not achieved, *P* = .0069), T2 (BDSR 41.2 months, PD 63.1 months, *P* = .0232) and N0 (BDSR 58.3 months, PD 81.7 months, *P* = .0210) cases. On the other hand, there were no significant differences between the BDSR and PD groups in T3 (BDSR 33.7 months, PD 37.0 months, *P* = .0788) and N1 (BDSR 31.3 months, PD 32.3 months, *P* = .3297) cases (Figure 2).

**Figure 3:**
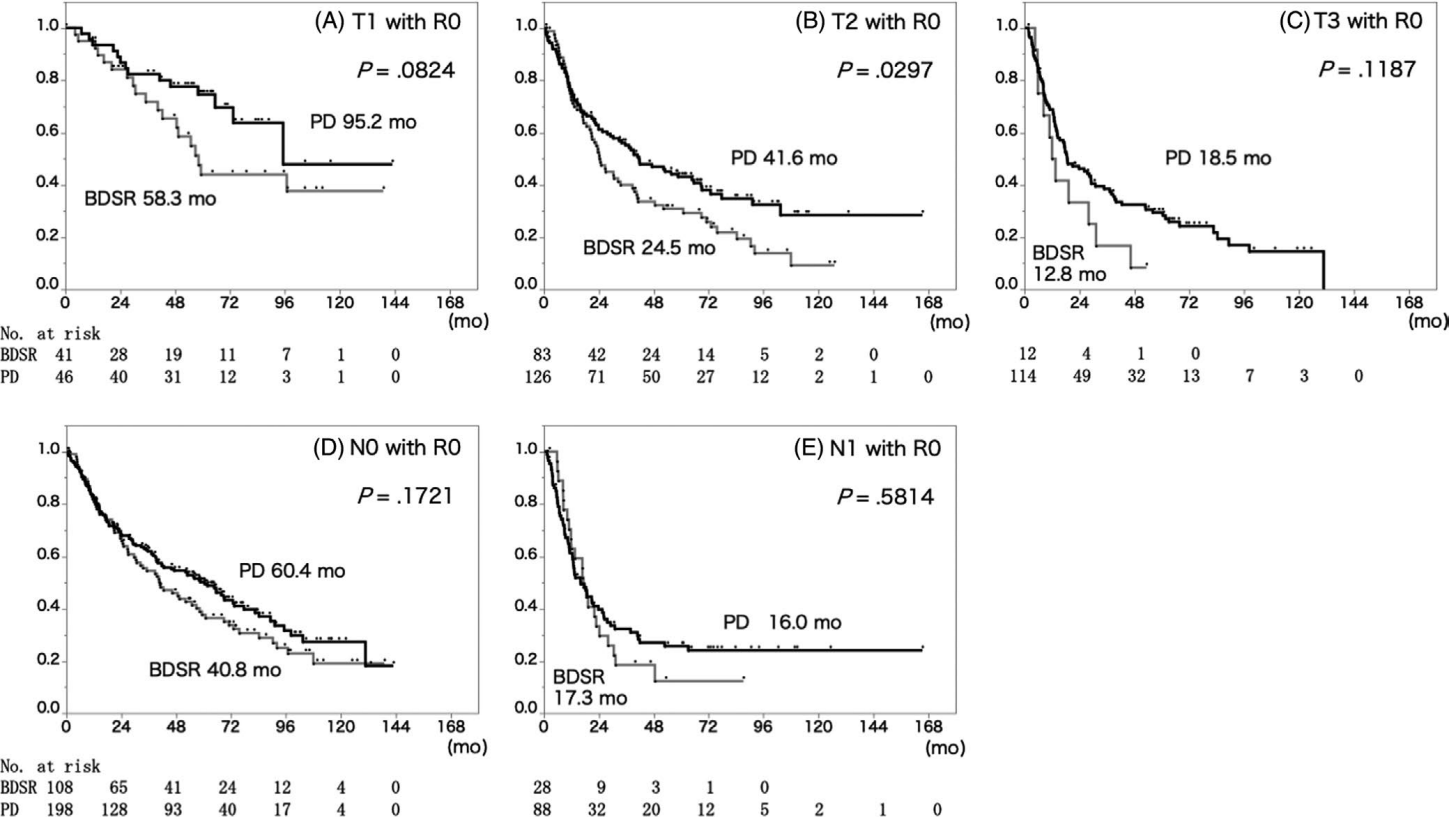
Relapse‐free survival curves of the patients with R0 resection stratified by T factor and N factor. Cox‐regression survival probability of (A) T1, (B) T2, (C) T3, (D) N0 and (E) N1 patients who received BDSR (gray line) or PD (black line). Though a significant difference was observed only in T2 cases, the PD group had a tendency of long relapse‐free survival time compared to the PDSR group

There was a tendency for longer RFS in the PD group when compared within R0 resected cases, but a significant difference was observed only within the T2 cases (BDSR 24.5 months, PD 41.6 months, *P* = .0297) (Figure 3).

**Figure 4:**
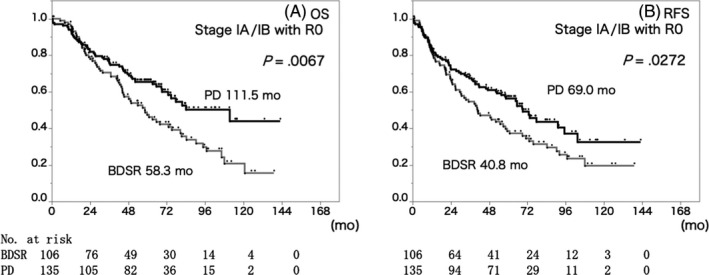
Overall survival (A) and relapse‐free survival (B) curve of the Stage IA/IB patients with R0 resection who received BDSR (gray line) or PD (black line). In Stage IA and IB cases, which were considered to be the best indication for BDSR, significantly longer MST and RFS were also observed in the PD group

In Stage IA and IB cases, which were the best indications for BDSR, the MST (BDSR 58.3 months, PD 111.5 months, *P* = .0067) and RFS (BDSR 40.8 months, PD 69.0 months, *P* = .0272) of the PD group were significantly better than those of the BDSR group even after R0 resection (Figure 4).

Comparing the incidence of recurrent site between the BDSR group and the PD group, local recurrence (BDSR 33.9%, PD 14.4%, *P* < .0001) and lymph node recurrence (BDSR 22.4%, PD 11.0%, *P* < .0001) was significantly higher in the BDSR group, but there was no difference in the rate of liver metastasis (BDSR 21.2%, PD 20.1%, *P* = .7283), peritoneal recurrence (BDSR 10.6%, PD 9.3%, *P* = .5921), and lung metastasis (BDSR 3.7%, PD 3.6%, *P* = .9549).

In univariate analysis, significant survival differences were observed in the stratification of pT (1, 2/3), pN (0/1), v (−/+), ly (−/+), ne (−/+), R (0/1, 2), tumor differentiation (well/other), the total number of retrieved lymph nodes (<13/≥13) and the operative procedures (BDSR/PD). Multivariate analysis using the Cox proportional hazard model showed that BDSR was an independent, poor prognostic factor (HR 1.57, 95% CI 1.20‐2.05, *P* = .0010) along with pT3, presence of lymph node metastasis (pN1), presence of (peri−) neural invasion (ne+) and tumor differentiation other than well (Table 2).

## 4 DISCUSSION

Achieving R0 resection, a curative surgery without cancer remnants, is considered critical during surgery for distal bile duct carcinoma.^12^, ^13^, ^14^, ^15^ Therefore, PD is usually preferred for distal BDCs, but in localized cholangiocarcinomas without pancreatic invasion or extension to the lower bile duct, R0 resection can be sometimes achieved with BDSR alone. In previous reports from a Korean group, there was no difference in prognosis between the PD and BDSR cases, while BDSR could also be applied if R0 resection was achievable.^7^, ^8^ In the current study, the R0 resection rate was significantly higher in the PD group (BDSR 55.5%, PD 68.4%, *P* = .0022) as in previous reports. Particularly in T3 and N1 cases, the ratio of R0 resection in the BDSR group was extremely low compared to the PD group and fell to below 50%. Therefore, the indication for BDSR is considered to be up to Stage IA and IB.

In spite of the high frequency of advanced cases in the PD group, OS and RFS were significantly longer in the PD group than in the BDSR group. Moreover, better long‐term outcomes were obtained in the PD group even within R0 resected cases with Stage IA and Stage IB, which were the best indications for performing BDSR. There was a possibility that the better survival in PD group was due to higher comorbidities in the BDSR group. However, RFS was also significantly longer in the Stage IA/IB patients when R0 resection was achieved, which indicated that better survival in the PD group was not due to comorbidities but due to cancer recurrence.

We also compared the differences in survival by country, but no differences were observed in trends that the PD group tend to have a longer survival than the BDSR group in all cases in both Japan and Korea (MST in Japan: BDSR 44.9 months, PD 64.9 months, *P* = .1716; MST in Korea: BDSR 37.6 months, PD 45.9 months, *P* = .0637), and even in R0 resected cases (MST in Japan: BDSR 63.0 months, PD 84.1 months, *P* = .2018; MST in Korea: BDSR 44.7 months, PD 48.1 months, *P* = .3613).

Our data showed that BDSR was an independent, poor prognostic factor along with pT3, presence of lymph node metastasis (pN1), presence of (peri−) neural invasion (ne+) and tumor differentiation other than well. These independent prognostic factors, except for the operative procedure, are similar to those previously reported in a meta‐analysis of distal cholangiocarcinoma.^16^

Insufficient dissection was presumed to be the cause of recurrence because the rates of local recurrence and lymph node metastasis after BDSR were significantly higher than those after PD. In fact, the number of retrieved lymph nodes in the BDSR group was significantly smaller than that in the PD group. Compared to PD, the tissues that were difficult to dissect in BDSR were the lymph nodes on the posterior and anterior surface of the pancreatic heads (No. 13, 17) and lymph nodes at the root of the superior mesenteric artery (SMA) (No. 14). The dissection of these tissues may contribute to the prophylaxis of recurrence and improvement in the prognosis of mid‐BDC. Kayahara et al reported that the incidence of lymph node metastasis in mid‐BDC patients was high particularly at the lymph nodes in the lower half of the hepatoduodenal ligament (No. 12a2, 12b2, 12p2), the lymph nodes on the posterior surface of the pancreatic heads (No. 13), and lymph nodes along the common hepatic artery (No. 8).^3^ They also reported that the incidence of cancer invasion to the pancreatic head nerve plexus was low, and therefore the dissection of pancreatic head nerve plexus may not be effective for the prevention of recurrence. Dissection of No. 12 and No. 8 lymph nodes during BDSR is technically possible to the same extent as PD; hence, there is a possibility that radical dissection of the No. 13 lymph nodes may have contributed mostly to the difference in prognosis between BDSR and PD.

Even when R0 resection was performed, the length of the distal bile duct stump margin after BDSR was inevitably shorter than that after PD. A surgical margin of <5 mm was reported to show a significantly higher incidence of local recurrence and poorer prognosis compared to cases with a surgical margin of 5 mm or greater.^17^ There may also be a possibility that the inadequate length of the distal surgical margin contributes to a poor prognosis.

This study has some limitations. In this study, R1 or R2 resection were significantly poorer prognostic factors in univariate analysis, but they were not significant factors in the multivariate analysis. Since BDSR remains an independent and significant poor prognostic factor, the R0 resection of BDSR may not be a true R0 resection because the bile duct, tissue around pancreas and lymph nodes remain to be dissected when PD is performed, and we cannot rule out the possibility of microscopic residual cancer in these tissues. In addition, because of no evidence of adjuvant therapy for bile duct cancer at that time, there were some cases who were unclear whether postoperative adjuvant therapy was performed, and who were unclear about the regimen and duration of adjuvant therapy. Therefore, the difference in the adjuvant treatment rate between two procedures and the difference in prognosis with or without adjuvant treatment had not been examined. Excluding the missing values about adjuvant therapy, 123 of 356 cases (34.5%) of R0 and R1 resected case received adjuvant chemotherapy, and there were no significant differences in the rate between the BDSR group and the PD group (29.0% vs 36.1%, *P* = .1708), and in R2 resected case, the receiving rate of chemotherapy was 40.0%, and there were no significant differences in the rate between the BDSR group and the PD group (33.3% vs 43.0%, *P* = .2534) and between Japan and Korea (42.2% vs 36.0%, *P* = .2360).

As the rate of cancer remnants at the proximal margin (HM), dissected margin (EM) and the distal margin (DM) was significantly higher in the BDSR group, there was a possibility of patient selection bias for the avoidance of aggressive surgery and the choice of a less invasive procedure at the cost of curability from the viewpoint of the preoperative characteristics, such as general conditions, comorbidities, impression of older physical appearance than actual age, and activities, that were not measurable by age or the degree of cancer progression surveyed in this study. In particular, cases with T3 might have a high possibility of selection bias. There may also be some bias in the ratio of patients receiving adjuvant chemotherapy and chemotherapy after recurrence if there was bias in the selection of the operative procedure by the patients' general condition or comorbidities. Comparative analysis should be performed in the future by adjusting patients' preoperative background and factors.

Certainly, compared to BDSR, PD is surgically invasive. A larger amount of intraoperative bleeding, longer operation time, higher incidence of pancreatic fistula, higher incidence of organ space SSI, and longer length of hospital stay after PD than those after BDSR were noted. However, as there was no difference in the mortality rate, PD should be selected unless it is extremely invasive and unavoidable due to the patient's general condition and comorbidities. Our data suggest that PD should be adopted as the standard procedure for mid‐BDCs and BDSR should be avoided even if R0 resection can be achieved.

## CONFLICT OF INTEREST

None declared.
